# Development of a Risk Model for Predicting Microalbuminuria in the Chinese Population Using Machine Learning Algorithms

**DOI:** 10.3389/fmed.2022.775275

**Published:** 2022-02-07

**Authors:** Wei Lin, Songchang Shi, Huibin Huang, Nengying Wang, Junping Wen, Gang Chen

**Affiliations:** ^1^Department of Endocrinology, Shengli Clinical Medical College of Fujian Medical University, Fujian Provincial Hospital, Fuzhou, China; ^2^Department of Critical Care Medicine, Shengli Clinical Medical College of Fujian Medical University, Fujian Provincial Hospital South Brance, Fujian Provincial Hospital Jinshan Branch, Fuzhou, China

**Keywords:** microalbuminuria, risk score, risk factors, machine learning, predicting

## Abstract

**Objective:**

Microalbuminuria (MAU) occurs due to universal endothelial damage, which is strongly associated with kidney disease, stroke, myocardial infarction, and coronary artery disease. Screening patients at high risk for MAU may aid in the early identification of individuals with an increased risk of cardiovascular events and mortality. Hence, the present study aimed to establish a risk model for MAU by applying machine learning algorithms.

**Methods:**

This cross-sectional study included 3,294 participants ranging in age from 16 to 93 years. R software was used to analyze missing values and to perform multiple imputation. The observed population was divided into a training set and a validation set according to a ratio of 7:3. The first risk model was constructed using the prepared data, following which variables with *P* <0.1 were extracted to build the second risk model. The second-stage model was then analyzed using a chi-square test, in which a *P* ≥ 0.05 was considered to indicate no difference in the fit of the models. Variables with *P* <0.05 in the second-stage model were considered important features related to the prevalence of MAU. A confusion matrix and calibration curve were used to evaluate the validity and reliability of the model. A series of risk prediction scores were established based on machine learning algorithms.

**Results:**

Systolic blood pressure (SBP), diastolic blood pressure (DBP), fasting blood glucose (FBG), triglyceride (TG) levels, sex, age, and smoking were identified as predictors of MAU prevalence. Verification using a chi-square test, confusion matrix, and calibration curve indicated that the risk of MAU could be predicted based on the risk score.

**Conclusion:**

Based on the ability of our machine learning algorithm to establish an effective risk score, we propose that comprehensive assessments of SBP, DBP, FBG, TG, gender, age, and smoking should be included in the screening process for MAU.

## Introduction

Microalbuminuria (MAU) is defined as a urinary albumin excretion of 20–200 mg/L in a spot urine test or 30–300 mg in a 24-h urine collection test (1). The presence of MAU represents an early manifestation of general endothelial damage, which can occur secondary to diabetes, hypertension, and coronary heart diseases (2, 3). Research has demonstrated that MAU is closely associated with stroke, myocardial infarction, coronary artery disease, and all-cause mortality (4). Several studies have also indicated that MAU is predictive of vascular disease, diastolic dysfunction, congestive heart failure, and hypertension (5–7). Hence, clinical screening and early identification of MAU remains especially important.

Advancements in proteomics technology such as protein separation, biological mass spectrometry, and bioinformatics have decreased the difficulty of examining proteome expression (8). Despite these advancements, there are still many drawbacks in the detection of urine albumin (8). The gold standard in chronic kidney disease (CKD) screening is the 24-h urine collection test; however, this method is difficult to implement on a large scale due to its inconvenience (2).

Therefore, in the present study, we aimed to establish and validate a risk model for early prediction of MAU using machine learning algorithms rather than the results of 24-h urine microalbumin tests. Application of risk scores derived using such a model would be more convenient for the monitoring and follow up of patients at higher risk for MAU.

## Methods

### Study Population

**Figure 1:**
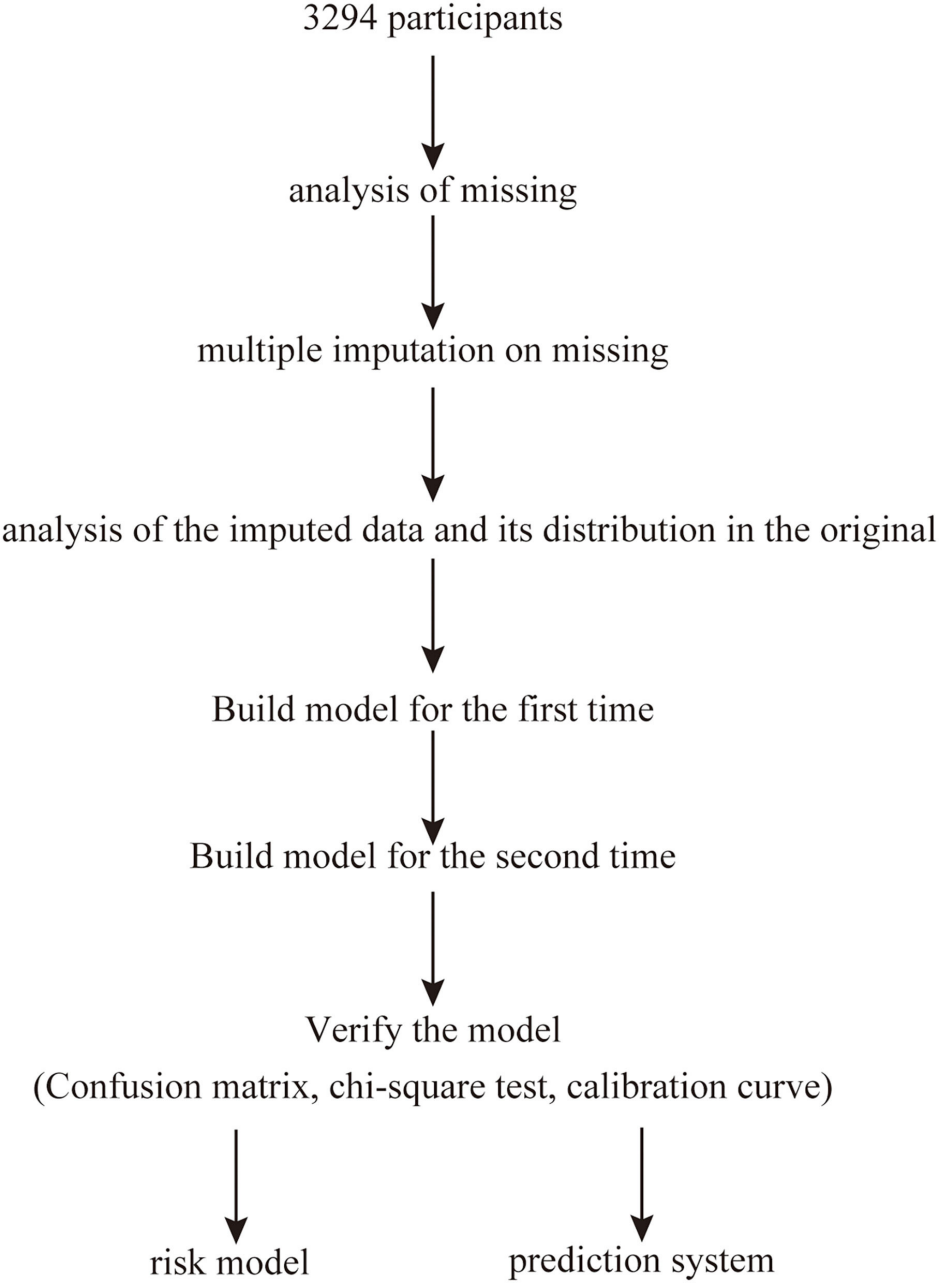
Study design flow.

This cross-sectional study was performed between June 2011 and January 2012 and included participants randomly selected using a clustered sampling technique (9), with probabilities proportionate to the size of the population in each cluster. All participants were from Ningde City in Fujian province in southeast China. Overall, 3,294 Chinese (age: 16–93 years) participants who had no cognitive dysfunction and were not pregnant participated in the survey. MAU was defined as a urinary albumin excretion of 20–200 mg/L and was assessed using a spot urine test (1, 4). The exclusion criteria were as follows: history of type-1 diabetes mellitus (DM), history of kidney disease or urinary albumin excretion ≥200 mg/L, and pregnancy. The study was performed in accordance with the Declaration of Helsinki and approved by the Ethics Committee of Fujian Provincial Hospital (approval No. K2009-12-020), and written informed consent was obtained from each participant. All investigators who were unaware of the study's aims or the characteristics of the participants received special training before the investigation. Figure 1 shows a flowchart describing patient selection.

### Data Collection

All participants were required to complete a standard self-reported questionnaire comprising 10 questions addressing age, sex, personal and family medical history, smoking and drinking habits, and so on.

Weight, height, and waist circumference (WC) were measured to the nearest 0.1 kg and 0.1 cm, respectively, by experienced nurses, with patients wearing light clothing and no shoes. WC was measured at the middle point between the costal margin and iliac crest. Systolic and diastolic blood pressures (SBP and DBP) were both measured twice using a standard OMRON auto-electronic sphygmomanometer, and the mean of the two readings was used for analysis.

Blood samples were collected after an 8- to 12-h overnight fast and were stored at −20°C until analysis. Participants were provided with oral and written instructions on the collection of urine samples and advised to postpone urine collection in case of urinary tract infection, fever, or menstruation, and to avoid heavy exercise as much as possible during the collection period. The blood samples were evaluated at the Laboratory of Ningde Municipal Hospital. Each blood sample was independently assessed by two qualified examiners. Blood glucose levels were determined using the glucose oxidase method (Sclavo, Siena, Italy). The automatic colorimetric method (Hitachi, Boehringer Mannheim) was used to determine total cholesterol (TC), total triglyceride (TG), and high-density lipoprotein cholesterol (HDL-C) levels. Low-density lipoprotein cholesterol (LDL-C) levels were calculated using the Friedewald formula.

Type-2 DM was defined as a fasting blood glucose (FBG) ≥7.0 mmol/L or 2-h postprandial blood glucose (PBG) ≥11.1 mmol/L, previous diagnosis of type-2 DM, or use of hypoglycemic medications (10). Hypertension was defined as SBP ≥140 mmHg and/or DBP ≥90 mmHg, or use of antihypertensive medications (11). The Homeostatic Model Assessment (HOMA) values for β-cell function and insulin resistance (IR) were determined using the following simplified equations: HOMA-IR = [fasting plasma insulin (FPI) × FPG]/22.5; HOMA-β = (20 × FPI)/(FPG−3.5) (12, 13). The following were used as indices of insulin secretion in the current study: insulinogenic index = (Ins_30_-Ins_0_)/(Gluc_30_-Gluc_0_), where Ins_y_ and Glu_y_ represent values at time (y:min) during the oral glucose tolerance test (OGTT) (14, 15).

### Statistical Analysis

All calculations were performed using R software (version 3.6.3 GUI 1.70 EI Capitan build, 7735).

The “vim” package for R software was used to analyze missing values and visualize the data. The “mice” package was used to perform multiple imputation on missing values (m = 5, method = “pmm,” maxit = 100, seed = 1,234). The imputed data and their distribution in the original dataset were analyzed and visualized using the “lattice” package.

The observed population was divided into a training set and a validation set according to a ratio of 7:3. The “glm” package was used to build the first risk model using the prepared data. Then, variables with *P* <0.1 were extracted to build the second risk model, also using the “glm” package. A chi-square test of the second-stage model was performed using the “anova” package, and a *P* ≥ 0.05 was considered to indicate no difference in the fit of the model. A confusion matrix was used to verify the accuracy of the model, and a calibration curve was constructed using the “calibrate” package. Values of *x* closer to *y* in the calibration curve were considered to indicate better calibration of the model. Variables with *P* <0.05 in the second-stage model were regarded as important features related to the prevalence of MAU. Graphical representations of the results were drawn using the “forestplot” package, and the risk score was established using a nomogram.

## Results

### Participant Characteristics

**Figure 2:**
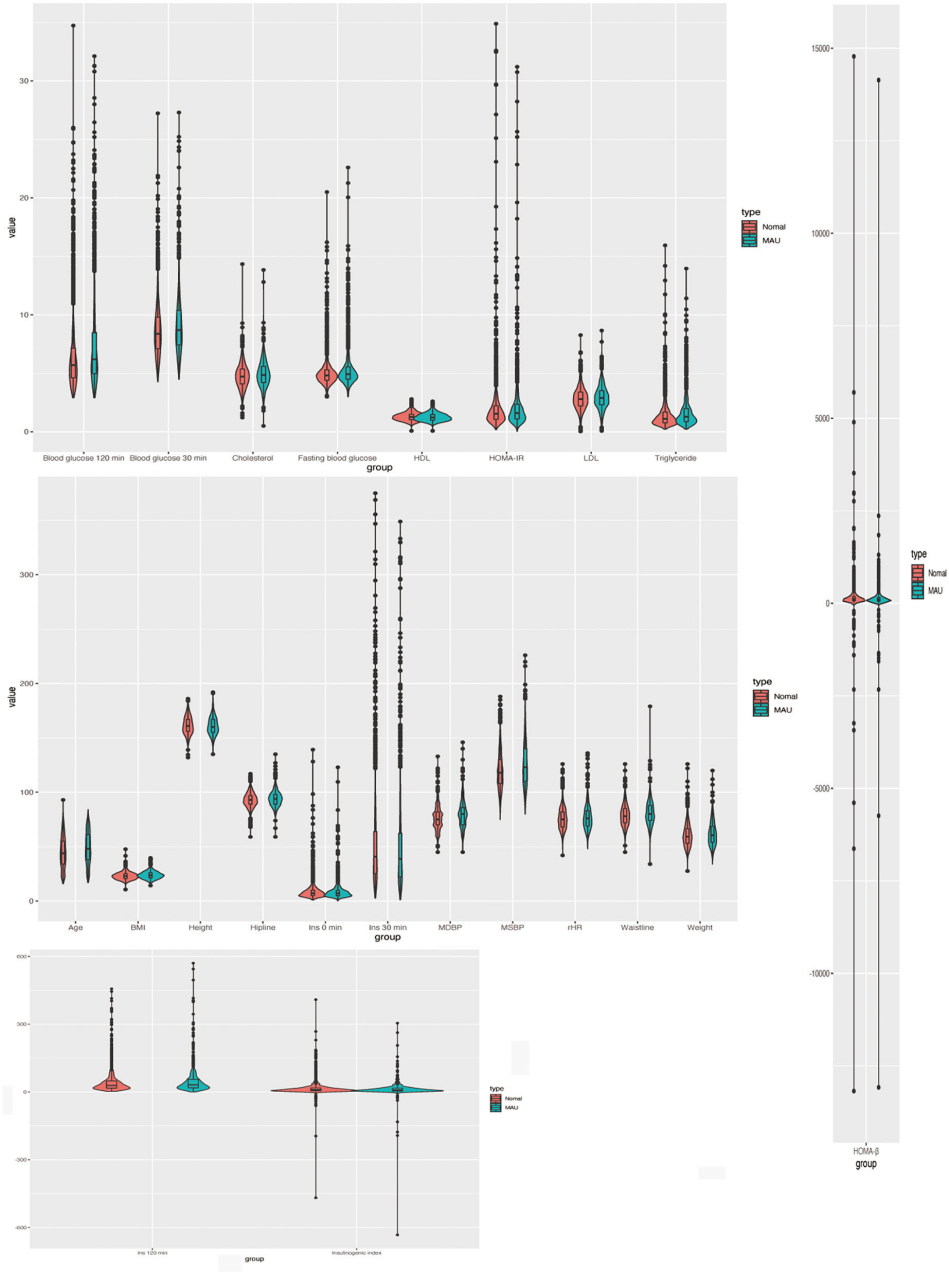
The distribution of characteristic of the participants.

The enrolled participants were categorized based on urinary albumin levels, gender, presence of hypertension/diabetes, and smoking and drinking habits. The study population comprised 3,294 study participants [men: 1,294 (39.3%); women: 2,000 (60.7%)]. The characteristics of the participants are shown in Tables 1, 2. A visual depiction of the distribution of these characteristics is shown in Figure 2.

**Table 1:** Characteristics of the study participants.

**Parameters**	**Men (%)**	**Women (%)**
Urinary albumin	444 (37.6%)	736 (62.4%)
Drinking	636 (73.4%)	231 (26.6%)
Smoker	*n* = 668 (97.1%)	*n* = 20 (2.9%)
Hypertension	*n* = 480 (40.3%)	*n* = 711 (59.7%)
Diabetes	*n* = 174 (44.5%)	*n* = 217 (55.5%)

**Table 2:** Laboratory data for the study participants.

	**Total**	**NMAU**	**MAU**
Ins_0 min (mmol/L)	7.12 (4.95 ~ 9.98)	7.14 (4.93 ~ 10.03)	7.09 (4.97 ~ 9.85)
Ins_30 min (mmol/L)	39.76 (24.07 ~ 63.43)	40.78 (25.25 ~ 63.82)	38.82 (22.20 ~ 62.35)
Ins_120 min (mmol/L)	29.88 (17.26 ~ 51.79)	28.90 (16.77 ~ 48.80)	31.80 (18.42 ~ 55.62)
Height (cm)	161 (155 ~ 167)	161 (156 ~ 167)	160 (155 ~ 167)
Weight (kg)	60 (53 ~ 67)	59.2 (53 ~ 67)	60.5 (54 ~ 69)
BMI	23.1 (21.0 ~ 25.4)	22.9 (20.7 ~ 25.1)	23.6 (21.4 ~ 26)
Waistline (cm)	79 (72 ~ 86)	78 (72 ~ 85)	80 (74 ~ 88)
Hipline (cm)	93 (89 ~ 98)	93 (89 ~ 97)	94 (89 ~ 98)
MSBP (mmHg)	120 (110 ~ 134)	118 (108 ~ 130)	123 (110 ~ 140)
MDBP (mmHg)	78 (70 ~ 85)	75 (70 ~ 82)	80 (70 ~ 86)
rHR (per min)	75 (69 ~ 83)	75 (68 ~ 82)	76 (69 ~ 83)
Bg_0 min (mmol/L)	4.87 (4.45 ~ 5.36)	4.82 (4.42 ~ 5.29)	4.93 (4.50 ~ 5.55)
Bg_30 min (mmol/L)	8.50 (7.24 ~ 10.00)	8.38 (7.10 ~ 9.80)	8.70 (7.43 ~ 10.38)
Bg_120 min (mmol/L)	5.88 (4.72 ~ 7.67)	5.70 (4.61 ~ 7.15)	6.20 (4.97 ~ 8.47)
Cholesterol (mmol/L)	4.79 (4.14 ~ 5.45)	4.73 (4.10 ~ 5.36)	4.86 (4.21 ~ 5.60)
Triglyceride (mmol/L)	1.17 (0.8 ~ 1.8)	1.11 (0.77 ~ 1.69)	1.28 (0.88 ~ 1.99)
HDL (mmol/L)	1.25 (1.02 ~ 1.50)	1.27 (1.04 ~ 1.50)	1.22 (1.00 ~ 1.48)
LDL (mmol/L)	2.83 (2.26 ~ 3.44)	2.79 (2.21 ~ 3.39)	2.90 (2.31 ~ 3.52)
Age (years)	46 (35 ~ 57)	44 (34 ~ 55)	48 (38 ~ 61)
HOMA-IR	1.56 (1.08 ~ 2.26)	1.54 (1.08 ~ 2.22)	1.60 (1.11 ~ 2.36)
HOMA-β	103.42 (63.76 ~ 168.21)	109.92 (68.19 ~ 175.45)	95.43 (56.77 ~ 153.86)
Insulinogenic-index	9.47 (4.71 ~ 17.36)	9.81 (5.03 ~ 17.69)	8.78 (4.26 ~ 16.64)

*NMAU, No microalbuminuria; MAU, microalbuminuria; Ins, Insulin releasing test; BMI, Body Mass Index; MSBP, mean systolic blood pressure; MDBP, mean diastolic blood pressure; rHR, resting heart rate; Bg, blood glucose; HDL, high-density lipoprotein; LDL, low-density lipoprotein; HOMA-IR, Homeostasis Model Assessment- insulin resistance; HOMA-β, Homeostasis Model Assessment of β-cell function*.

*Data are shown as the median (interquartile range)*.

### Analysis of Missing Values

**Figure 3:**
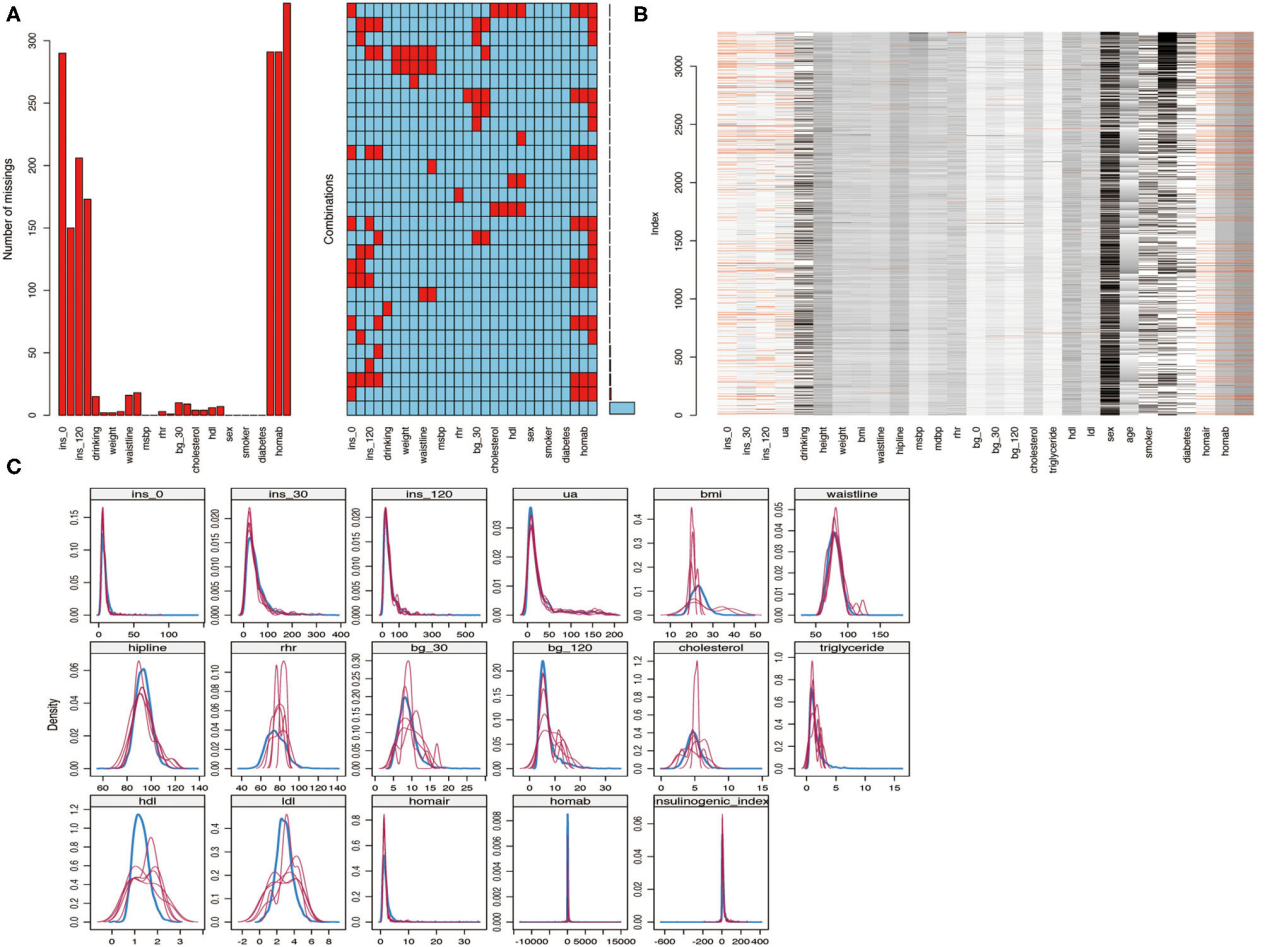
**(A,B)** Analysis of missing values; **(C)** Multiple imputed data and its distribution in the original data.

Twenty-eight observation indices were analyzed for missing values. The insulinogenic index represented the index with the most missing values, accounting for 10% (*n* = 330), followed by HOMA-IR and HMOA-β, which accounted for <10%. An analysis of trends in the distribution of missing values indicated that they were randomly distributed, conforming to the missing-at-random (MAR) assumption (Figures 3A,B). The “mice” package was used to perform multiple imputation on data with missing values (m = 5, method = “pmm,” maxit = 100, seeds = 1,234). The imputed data and their distribution in the original dataset are shown in Figure 3C.

### Risk Model for MAU

**Figure 4:**
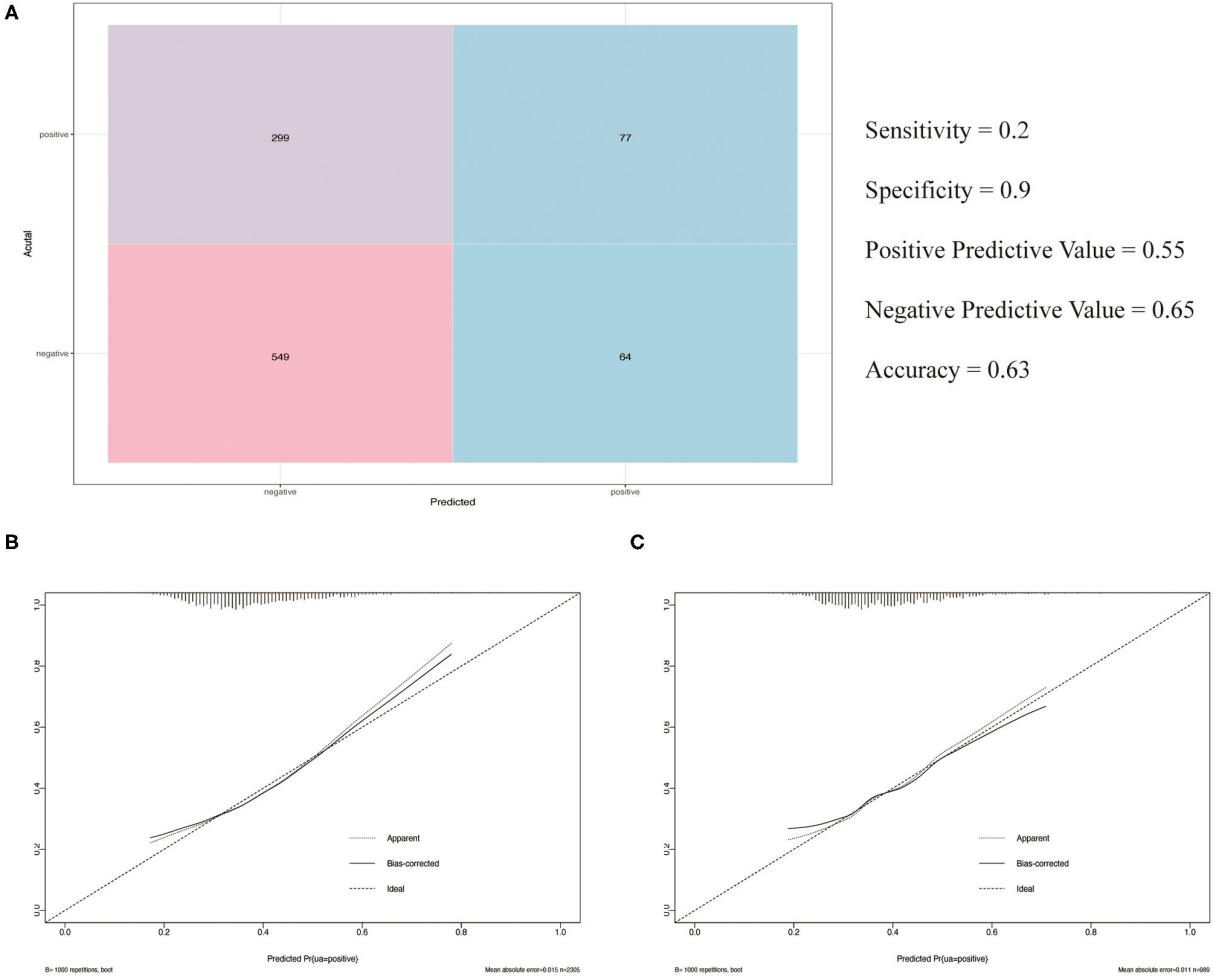
**(A)** Confusion matrix; **(B)** The calibration curve of the training set; **(C)** The calibration curve of the validation set.

**Figure 5:**
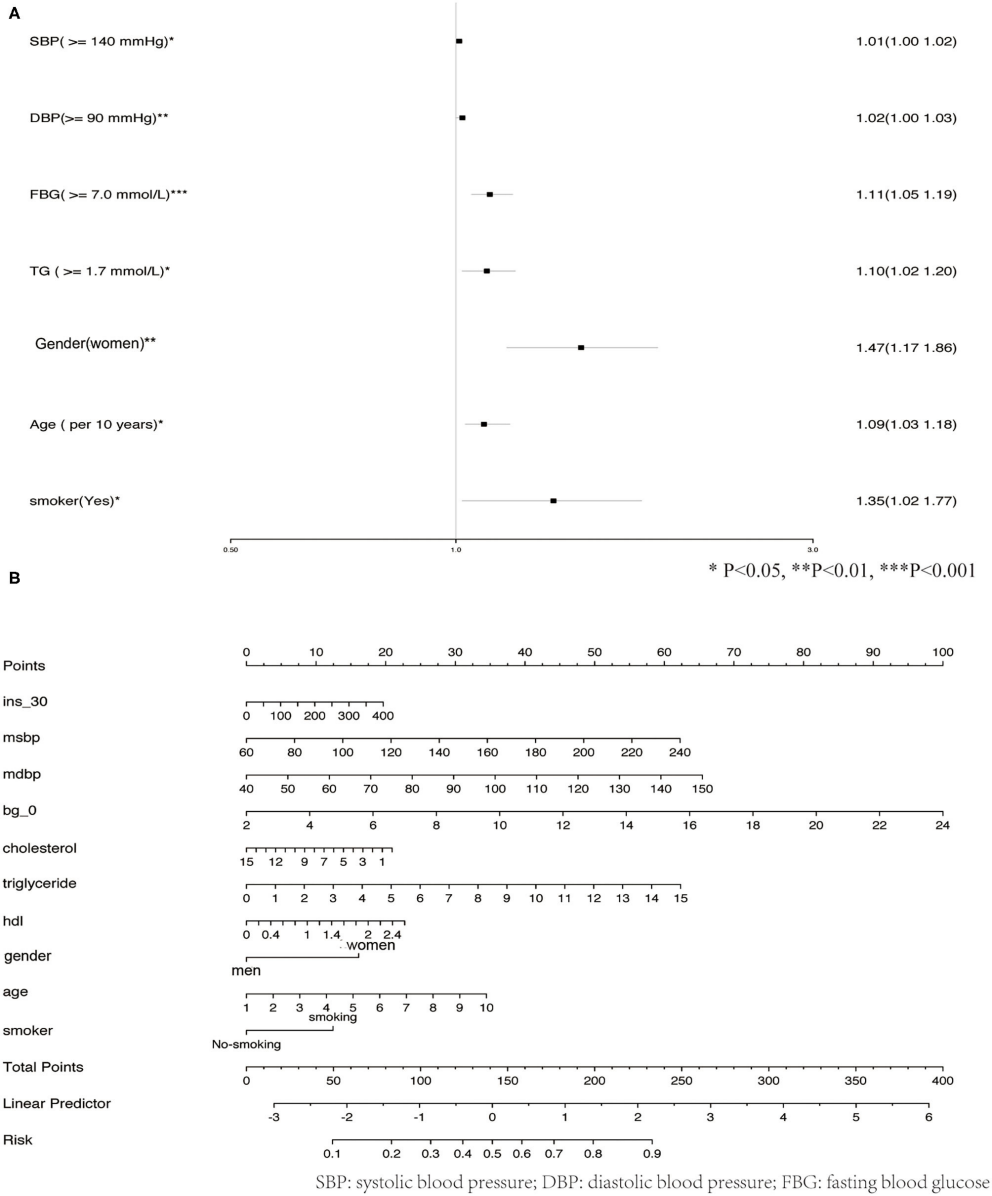
**(A)** Risk model of the MAU. **(B)** Risk score to predict the risk of MAU.

The observed population was divided into a training set and a validation set according to a ratio of 7:3 (training set: 2,305; validation set: 989). The 2,305 cases in the training set were used to build the first predictive model, which is described in Table 3. Significant factors in this model (P <0.10) included SDP, DBP, Bg_0 min, TC, TG level, HDL level, gender, age, and smoking. Logistic model fitting was performed again after extracting the variables with *P* <0.10. The second-stage model was then evaluated using a chi-square test, confusion matrix, and a calibration curve. The specificity of the model in the verification set reached as high as 0.9, with an accuracy of 0.63. The positive and negative predictive values were 0.55 and 0.65, respectively (Figure 4A). In the calibration curve, values of *x* remained close to *y*, indicating good calibration in both the training and validation sets (Figures 4B,C). Based on a *P* <0.05, important features related to the incidence of MAU in the second-stage model included mean SBP, mean DBP, FBG, TC, TG level, HDL, gender, age, and smoking (Figure 5A).

**Table 3:** Initial risk model.

	**Estimate**	**Std. error**	***P*-value**
Ins_0 min (mmol/L)	−2.20e-03	1.81e-02	0.90
Ins_30 min (mmol/L)	2.65e-03	1.28e-03	0.04^**^
Ins_120 min (mmol/L)	−4.40e-04	1.07e-01	0.68
Drinking (Yes)	−3.02e-03	1.17e-02	0.98
Height (cm)	−1.47e-02	2.49e-02	0.55
Weight (kg)	3.01e-02	3.18e-02	0.34
BMI	−7.31e-02	8.22e-02	0.37
Waistline (cm)	7.52e-03	7.96e-03	0.34
Hipline (cm)	−8.49e-03	1.10e-02	0.44
MSDP (≥140 mmHg)	6.61e-03	3.66e-03	0.07^*^
MDBP (≥90 mmHg)	1.23e-02	5.74e-03	0.03^*^
rHR (per min)	−1.69e-03	4.48e-03	0.71
Bg_0 min (≥7 mmol/L)	1.43e-01	6.41e-02	0.03^**^
Bg_30 min (mmol/L)	−3.31e-02	2.74e-02	0.23
Bg_120 min (mmol/L)	3.45e-02	2.63e-02	0.19
Cholesterol (mmol/L)	−2.68e-01	1.56e-01	0.09^*^
Triglyceride (≥1.7 mmol/L)	1.94e-01	7.44e-02	0.01^**^
HDL (mmol/L)	4.65e-01	2.08e-01	0.03^*^
LDL (mmol/L)	2.56e-01	1.60e-01	0.11
Gender (women)	4.61e-01	1.51e-01	0.001^***^
Age (years)	7.47e-02	4.02e-02	0.06^*^
Smoking (Yes)	2.92e-01	1.43e-01	0.04^**^
Hypertension (Yes)	1.70e-01	1.19e-01	0.15
Diabetes (Yes)	−2.93e-01	2.14e-01	0.17
HOMA-IR	−2.39e-02	6.11e-02	0.70
HOMA-β	−6.47e-05	6.98e-05	0.35
Insulinogenic index	−3.16e-03	1.93e-03	0.10

*Ins, Insulin releasing test; BMI, Body Mass Index; MSBP, mean systolic blood pressure; MDBP, mean diastolic blood pressure; rHR, resting heart rate; Bg, blood glucose; HDL, high-density lipoprotein; LDL, low-density lipoprotein; HOMA-IR, Homeostasis Model Assessment- insulin resistance; HOMA-β, Homeostasis Model Assessment of β-cell function*.

* *P < 0.10*,

** *P < 0.05*,

*** *P < 0.01*.

### Development of an MAU Risk Score

Given their significant relationship with MAU based on our analysis of the second-stage model, the following variables were used to develop the risk prediction system: mean SBP, mean DBP, FBG, TC, TGs, HDL, gender, age, and smoking. Figure 5B shows how total risk scores for MAU are calculated.

## Discussion

MAU is an early marker of diabetic kidney disease (DKD) (2), cardiovascular disease, and renal risk (1). Accounting for ~50% of end-stage kidney disease (ESKD) cases in the developed world (16), DKD has a major effect on global healthcare costs and resources (2). Estimates indicate that the prevalence of MAU among patients with type-2 DM in the Asia-Pacific region ranges from 17.0 to 18.2%, while severe albuminuria and reduced estimated glomerular filtration rate (eGFR) are observed in 2.1–14.1 and 15.3–61.6% of patients, respectively (17, 18). These statistics highlight the importance of screening, early detection, and prevention efforts to reduce the overall impact of MAU.

Given that diabetic glomerulopathy can be only be diagnosed definitively via a kidney biopsy, few studies to date have investigated methods for predicting MAU (3), making it difficult to perform a detailed analysis of MAU risk (19). DKD may be present long before the patient develops traditional indications for a kidney biopsy (20). Careful screening and prediction using the risk score developed in our study may allow for early detection of MAU without the need for a kidney biopsy.

In contrast to previous findings, DM was not identified as an independent factor influencing MAU risk in the current study. This inconsistency may be related to the low proportion of patients with DM among our participants (11.9%). In the surveyed population, elevated FBG and PBG were more prevalent than DM, suggesting that diabetes had not been identified in some patients. However, the risk associated with elevated FBG was as high as 1.11 [odds ratio (OR): 1.11, 1.05–1.19], indicating that elevated blood glucose was still an independent risk factor for MAU.

Increased intraglomerular capillary pressure, which is related to systemic blood pressure as well as pre- and post-glomerular resistance, is the most important determinant of MAU (21, 22). Previous studies have reported that blood pressure is closely associated with albuminuria in patients with hypertension and in the general population (23–25). A study conducted among the Japanese population demonstrated that SBP exhibited an independent positive correlation with MAU (21). Another Japanese study indicated that both systolic hypertension and hyperglycemia were independent risk factors for MAU, in accordance with our findings (21). Saadi et al. have also observed that SBP and DBP are significantly higher patients with MAU than in the general population (26).

One study conducted in China reported that, for each 10 mg increment in 24-h urinary microalbumin excretion within the normal range, the odds of significantly elevated TG levels increased by 41% (24). Our analysis indicated that, when compared with the normal TG range, abnormally elevated TG levels increase the risk of MAU by a factor of 1.10 [odds ratio (OR): 1.10, 1.02–1.20].

Although Ge et al. (24) observed no significant difference in gender in 24-h urinary microalbumin excretion in a study of Chinese adults, our results are in contrast to these findings. Our analysis identified gender as an important feature influencing the incidence of MAU (OR: 1.47, 1.17–1.86). In Japan, the albumin/creatinine ratio is higher in women and older adults than in men and younger individuals, respectively, but this is not true for the albumin concentration (21). Our findings also indicated that, for each 10-year increment, the odds of TG elevation significantly increased by 9% (OR: 1.09, 1.03–1.18).

Several previous studies have reported that MAU is related to smoking (27–29) and obesity (30–32), while others have noted the influence of race and region on MAU prevalence (33–35). Our study suggests that smoking is indeed an important feature affecting the prevalence of MAU (OR: 1.35, 1.02–1.77), while no such relationship was observed for obesity. However, despite appropriate calibration of the model, the low incidence of obesity among our patients may have influenced our results.

To our knowledge, the current study is the first to establish a risk score for MAU using a large sample of patients, to establish such a model using multiple imputation to account for missing data, and to utilize chi-square and logistic fitting for double-verification of model quality. Nonetheless, the study also had some potential limitations, including relatively limited variations in race and region. Furthermore, this was a single-center and cross-sectional study, necessitating verification of our model in multicenter studies with long-term follow-up periods.

In conclusion, based on our analysis using machine learning algorithms, we propose that comprehensive assessments of SBP, DBP, FBG, TG, gender, age, and smoking be included in the screening process for MAU. The risk score established in the present study may allow clinicians and patients to initiate early interventions that can delay or prevent the development of MAU.

## Data Availability Statement

The raw data supporting the conclusions of this article will be made available by the authors, without undue reservation.

## Ethics Statement

The studies involving human participants were reviewed and approved by Fujian Provincial Hospital Ethics Committee. The patients/participants provided their written informed consent to participate in this study.

## Author Contributions

WL and SS performed the statistical analysis and wrote the first draft of the manuscript. HH, NW, and JW reviewed, edited, critically revised the manuscript, approved the final version of the manuscript, and interpreted the data. JW and GC designed the study. GC had full access to all the data in the study and takes responsibility for the integrity of the data and the accuracy of the data analysis. All authors contributed to the article and approved the submitted version.

## Funding

This study was supported by the National Key Research and Development Program of China (2018YFC2001100-5), the Natural Science Foundation of China (82070878), and the Natural Science Foundation of Fujian Province (Grant No. 2020J011068).

## Conflict of Interest

The authors declare that the research was conducted in the absence of any commercial or financial relationships that could be construed as a potential conflict of interest.

## Publisher's Note

All claims expressed in this article are solely those of the authors and do not necessarily represent those of their affiliated organizations, or those of the publisher, the editors and the reviewers. Any product that may be evaluated in this article, or claim that may be made by its manufacturer, is not guaranteed or endorsed by the publisher.
